# Supplementary material for: Limits on amplifiers of natural selection under death-Birth updating
Source: PLoS Comput Biol. 2020 Jan 17;16(1):e1007494. doi: 10.1371/journal.pcbi.1007494 (PMC6968837; doi:10.1371/journal.pcbi.1007494)
Supplement: S1 Appendix — (PDF) [file pcbi.1007494.s001.pdf]

Supplementary Information: Limits on amplifiers of natural selection under death-Birth updating

Josef Tkadlec[a, 1], Andreas Pavlogiannis[b, 1], Krishnendu Chatterjee[a], and Martin A. Nowak[c, ⋆]

[a]IST Austria, A-3400 Klosterneuburg, Austria
[b]Lab for Automated Reasoning and Analysis, EPFL, CH-1015 Lausanne, Switzerland
[c]Program for Evolutionary Dynamics, Department of Organismic and Evolutionary Biology, Department of Mathematics, Harvard University, Cambridge, MA 02138, USA
[1]J.T. and A.P. contributed equally to this work.
[⋆]To whom correspondence should be addressed. E-mail: martin_nowak@harvard.edu

Contents

1 Organization **1**

2 Model **1**

3 Results **4**

4 Proofs **5**
4.1 Theorems on dB updating 5
4.2 Theorems on δ-dB updating 8

5 Further directions **9**

1 Organization

This is supplementary information to the paper Limits on amplifiers of natural selection under death-Birth updating. The organization of this text is as follows. In Section 2, we formally introduce the model of Moran process on graphs with Birth-death (Bd) updating, death-Birth (dB) updating and δ-dB updating. We also introduce amplifiers of selection and classify them by universality and strength. In Section 3 we list our theoretical results and their significance. in Section 4 we present formal proofs. Finally, in Section 5 we list several interesting open questions for future research.

2 Model

Here we present the model of Moran process on graphs.

Population structure. In evolutionary graph theory, a population structure is represented by a graph that has N sites (nodes), some of which are connected by edges. Each site is occupied by a single individual. The edge from node u to node v represents that the individual at node u can replace the individual at node v.

Directions and weights. The edges could be undirected (two-way) or directed (one-way) and they could be weighted. Formally, for a pair of nodes u, v, the weight of an edge (u, v) is denoted by $w_{u,v}$. If the nodes u, v are not connected then $w_{u,v} = 0$. In the special case of unweighted graphs, each edge is considered to have weight 1. In the special case of undirected graphs, each edge is two-way. In the most general case of directed graphs with weighted edges, two nodes u, v could be interacting in both directions with different weights $w_{u,v} \neq w_{v,u}$. We don't allow self-loops, that is, $w_{u,u} = 0$ for each node u.

Mutant initialization. Initially, each site is occupied by a single resident with fitness 1. Then a single mutant with fitness r appears at a certain node. This initial mutant node can be selected uniformly at random (*uniform initialization*) or with probability proportional to the turnover rate of each node (*temperature initialization*). Unless specified otherwise, we assume that the initialization is uniform and that the mutation is advantageous ($r > 1$).

Moran dB and Bd updating. Once a mutant has appeared, some version of Moran process takes place. Moran process is a discrete-time stochastic process. At each step, one individual is replaced by a copy of another (neighbouring) individual, hence the population size remains constant. Denote by $f(v)$ the fitness of the individual at node v. The two prototypical updatings are:

- Moran death-Birth (dB) updating. An individual v is selected uniformly at random for death. The individuals at the neighbouring sites then compete for the vacant spot. Specifically, once v is fixed, an individual u is selected for placing a copy of itself on v with probability proportional to $f(u) \cdot w_{u,v}$. Note that fitness of an individual doesn't play a role in the death step (thus "d" is lower case) but it does play a role in the birth step (thus "B" is upper case).

- Moran Birth-death (Bd) updating. An individual u is selected for reproduction with probability proportional to its fitness $f(u)$. Then it replaces a random neighbor. Specifically, once u is fixed, an individual v is replaced by a copy of u with probability proportional to $w_{u,v}$.

Mixed δ-dB updating. The two regimes dB and Bd can be understood as two extreme points of a spectrum. We also consider mixed updating where some steps of the process follow the dB updating while the other ones follow Bd updating. Generally, given a $\delta \in [0, 1]$, a δ-dB updating is an update rule in which each step is a dB event with probability δ and a Bd event with probability $1 - \delta$, independently of all the other steps. With this notation, a 1-dB updating is the same as (pure) dB updating and 0-dB updating is the same as (pure) Bd updating.

Fixation probability. Given a graph G, $r > 1$ and $\delta \in [0, 1]$, we denote by $\mathrm{fp}^{\delta}(G, r)$ the fixation probability of a δ-dB updating, when the first mutant is initialized uniformly at random. The complement, that is the probability that the evolutionary trajectory goes extinct, is denoted by $\mathrm{ep}^{\delta}(G, r) = 1 - \mathrm{fp}^{\delta}(G, r)$. Specifically, for $\delta = 1$ we denote the fixation (resp. extinction) probability under pure dB updating by $\mathrm{fp}^{\mathrm{dB}}(G, r)$ (resp. $\mathrm{ep}^{\mathrm{dB}}(G, r)$) and similarly for the pure Bd updating which corresponds to $\delta = 0$.

Fixation probability on well-mixed populations. When studying the effect of population structure on the fixation probability, our baseline is the fixation probability on a well-mixed population of the same size. A well-mixed population is modelled by a complete (unweighted) graph K_N, without self-loops. Under pure dB and Bd updating there are exact formulas for fixation probability [1, 2]:

$$\mathrm{fp}^{\mathrm{dB}}(K_N, r) = \frac{N-1}{N} \cdot \frac{1-\frac{1}{r}}{1-\frac{1}{r^{N-1}}} \quad \text{and} \quad \mathrm{fp}^{\mathrm{Bd}}(K_N, r) = \frac{1-\frac{1}{r}}{1-\frac{1}{r^N}}.$$

For δ-dB updating, no analogous formula is known but numerical computations for various values of N and r show that $\mathrm{fp}^{\delta}(K_N, r)$ is essentially indistinguishable from the linear interpolation

$$\widehat{\mathrm{fp}}^{\delta}(K_N, r) = \delta \cdot \mathrm{fp}^{\mathrm{dB}}(K_N, r) + (1-\delta) \cdot \mathrm{fp}^{\mathrm{Bd}}(K_N, r)$$

between $\mathrm{fp}^{\mathrm{dB}}(K_N, r)$ and $\mathrm{fp}^{\mathrm{Bd}}(K_N, r)$ (see Fig. 2 from the main text). Therefore, in δ-dB updating we use $\widehat{\mathrm{fp}}^{\delta}(K_N, r)$ as the baseline comparison.

Amplifiers of selection. Given $r > 1$, some population structures enhance the fixation probability of mutants, compared to the well-mixed population, whereas others decrease it. We refer to the former ones as *amplifiers of selection* and to the latter ones as *suppressors of selection.* Formally, given a graph G_N with N nodes and some $r > 1$, we say that G_N is an *r-amplifier under dB updating* if $\mathrm{fp}^{\mathrm{dB}}(G_N, r) > \mathrm{fp}^{\mathrm{dB}}(K_N, r)$, where K_N is a complete graph that represents a well-mixed population. If G is an r-amplifier under dB updating for all $r > 1$, we call it *universal.* In contrast, graphs that amplify only for some range of values $r \in (1, r^{\star})$ are called *transient.* Similarly, we say that G_N is an *r-amplifier under Bd updating* if $\mathrm{fp}^{\mathrm{Bd}}(G_N, r) > \mathrm{fp}^{\mathrm{Bd}}(K_N, r)$ (note that the baseline is the complete graph K_N under Bd updating) and, for a fixed $\delta \in [0, 1]$, we say that G_N is an *r-amplifier under δ-dB updating* if $\mathrm{fp}^{\delta}(G_N, r) > \widehat{\mathrm{fp}}^{\delta}(K_N, r)$.

Classification of amplifiers by strength: Implied scale of fitness. Amplifiers can be further classified by strength [3]. We single out bounded amplifiers, linear amplifiers, quadratic amplifiers and super amplifiers. The intuition behind the classification is that, in the limit of large population size, fixation probability can often be written as $1 - 1/\operatorname{isf}(r)$ for a suitable function $\operatorname{isf}(r)$ of r. For instance, for large well-mixed population we have $\operatorname{isf}(r) = r$ (under any of dB, Bd, δ-dB updating) and for large Star graphs under Bd updating we have $\operatorname{isf}(r) = r^2$. The extent to which a large population structure G distorts this fixation probability can thus be classified by looking at the function $\operatorname{isf}(r)$.

Formally, given a family of graphs $\{G_N\}_{N=1}^{\infty}$ of increasing population size, the *implied scale of fitness* of the family is a function $\operatorname{isf}(r)\colon (1, \infty) \to \mathbb{R}$ such that

$$\lim \inf_{N\to\infty} \mathrm{fp}^{\mathrm{dB}}(G_N, r) = 1 - 1/\operatorname{isf}(r).$$

We say that the family is

1. an (at most) *bounded amplifier* if $\operatorname{isf}(r) \le r + c_0$ for some constant c_0.

2. an (at least) *linear amplifier* if $\operatorname{isf}(r) \ge c_1 r + c_0$ for some constants $c_1 > 1$, c_0.

3. an (at least) *quadratic amplifier* if $\operatorname{isf}(r) \ge c_2 r^2 + c_1 r + c_0$ for some constants $c_2 > 0$, c_1, c_0.

4. a *super amplifier* if $\mathrm{isf}(r) = \infty$ for all $r > 1$.

These definitions naturally carry over to Bd updating and δ-dB updating.

Remark on the regimes considered. We intentionally restrict our attention to the following regimes:

1. $r > 1$. If $r = 1$ then $\mathrm{fp}^{\delta}(G_N, r) = 1/N$, regardless of the population structure. If $r < 1$ then $\mathrm{fp}^{\delta}(G_N, r) < 1/N \to_{N\to\infty} 0$ for any G_N. Thus we focus on $r > 1$.

2. Uniform initialization. For dB updating, the notions of uniform and temperature initialization coincide, since every node is, on average, selected for death and replaced equally often. Thus we focus on uniform initialization only.

3. No self-loops. For dB updating, self-loops are not biologically realistic: An individual who has just died can not replace itself. Thus we consider graphs with possibly directed and/or weighted edges but without self-loops.

3 Results

Here we formally state our theorems and their consequences. The first two theorems concern pure dB updating, the other two concern mixed δ-dB updating.

Theorem 1 (All dB amplifiers are bounded)**.** *Fix $r > 1$. Then for any graph G_N (possibly with directed and/or weighted edges) we have $\mathrm{fp}^{\mathrm{dB}}(G_N, r) \le 1 - \frac{1}{r+1}$.*

Theorem 2 (All dB amplifiers are transient)**.** *Fix a non-complete graph G_N (possibly with directed and/or weighted edges). Then there exists $r^\star > 1$ such that for all $r > r^\star$ we have $\mathrm{fp}^{\mathrm{dB}}(G_N, r) < \mathrm{fp}^{\mathrm{dB}}(K_N, r)$, where K_N is the complete graph on N vertices. In particular, we can take $r^\star = 2N^2$.*

Theorem 3 (All δ-dB amplifiers are at most linear)**.** *Fix $r > 1$ and $\delta \in (0, 1]$. Then for any graph G (possibly with directed and/or weighted edges) we have $\mathrm{fp}^{\delta}(G, r) \le 1 - \frac{1}{(r/\delta)+1}$.*

Theorem 4 (All δ-dB amplifiers are transient)**.** *Fix a non-complete graph G on N vertices (possibly with directed and/or weighted edges) and $\delta \in (0, 1]$. Then there exists $r^\star > 1$ such that for all $r > r^\star$ we have $\mathrm{fp}^{\delta}(G, r) < \widehat{\mathrm{fp}}^{\delta}(K_N, r)$, where K_N is the complete graph on N vertices.*

Significance of our results. Here we list the implications of our results.

1. Theorem 1 implies that, under dB updating, no unweighted graph is a universal amplifier and a weighted graph can only be a universal amplifier if it is a weighted version of the complete graph K_N.

2. Theorem 2 implies that, under dB udpating, there are no superamplifiers and no quadratic or even linear amplifiers.

3. Theorem 3 is a δ-dB analogue of Theorem 1. It implies that, compared to the baseline given by a weighted average between $\mathrm{fp}^{\mathrm{dB}}(K_N, r)$ and $\mathrm{fp}^{\mathrm{Bd}}(K_N, r)$, no unweighted graph is a universal amplifier and a weighted graph can only be an r-universal amplifier if it is a weighted version of K_N.

4. Theorem 4 is a δ-dB analogue of Theorem 2. It implies that if $\delta > 0$ (i.e. we don't have pure Bd updating) then there are no quadratic amplifiers and no superamplifiers. For $\delta = 1$ (pure dB updating), the bound coincides with the one given in Theorem 2. For $\delta = 0$ (pure Bd updating), the bound is vacuous (in the limit $\delta \to 0$ it simplifies to $\mathrm{fp}^{\mathrm{Bd}}(G, r) \le 1$) which is in alignment with the existence of quadratic amplifiers and superamplifiers under (pure) Bd updating.

4 Proofs

Our proofs rely on Jensen's inequality. For reference purposes, we state it here. Essentially, given a convex (or concave) function f and several real numbers $x_1, \ldots, x_k$, Jensen's inequality bounds the (weighted) average of values $f(x_1), \ldots, f(x_k)$ by the value that f takes at the (weighted) average of $x_1, \ldots, x_k$.

Claim (Jensen's inequality)**.** Let $a_1, \ldots, a_n$ be non-negative real numbers that sum up to 1 and let f be a real continuous function. Then

- If f is convex then
$$\sum_{i=1}^{k} a_i \cdot f(x_i) \ge f\left(\sum_{i=1}^{k} a_i \cdot x_i\right)$$
- If f is concave then
$$\sum_{i=1}^{k} a_i \cdot f(x_i) \le f\left(\sum_{i=1}^{k} a_i \cdot x_i\right)$$

4.1 Theorems on dB updating

The key to proving our theorems on dB updating is the following lemma that gives an upper bound on the fixation probability $\mathrm{fp}^{\mathrm{dB}}(G, r)$ on an arbitrary graph (possibly with directed and/or weighted edges), in terms of the average in-degree d and the relative fitness $r > 1$ of the mutant. Recall that given a graph G and its node v, the *in-degree* of v is the number of nodes u for which there is an edge (u, v). If G is undirected then the in-degree of a node is the same as the degree (the number of neighbors). For any graph G, the average in-degree is the same as the average out-degree (and as the average degree if G is undirected).

Lemma 1. Fix $r > 1$ and let G be a graph (possibly with directed and/or weighted edges) with average out-degree d. Then
$$\mathrm{fp}^{\mathrm{dB}}(G, r) \le \frac{d \cdot r}{d \cdot r + d + r - 1}.$$

Proof. Denote by u the initial node occupied by the mutant and recall that $\mathrm{ep}^{\mathrm{dB}}(u)$ is the extinction probability under dB updating if the initial mutant appears at u. Then $\mathrm{ep}^{\mathrm{dB}}(G, r) = \frac{1}{N} \sum_u \mathrm{ep}^{\mathrm{dB}}(u)$.

Denote by $\mathcal{E}^-(u)$ (resp. $\mathcal{E}^+(u)$) the event that in the next step of the dB updating the number of mutants decreases (resp. increases) and by $p^-(u)$ (resp. $p^+(u)$) the corresponding probability. Note that if neither of $\mathcal{E}^-(u)$, $\mathcal{E}^+(u)$ happens, the set of nodes occupied by the mutants stays

the same, and if $\mathcal{E}^-(u)$ happens before $\mathcal{E}^+(u)$, the mutants go extinct. Therefore the extinction probability $\mathrm{ep}^{\mathrm{dB}}(u)$ starting from a configuration with a single mutant at node u satisfies

$$\mathrm{ep}^{\mathrm{dB}}(u) \geq \frac{p^-(u)}{p^-(u)+p^+(u)} = \frac{1}{1+\frac{p^+(u)}{p^-(u)}}.$$

We now compute $p^-(u)$ and $p^+(u)$. The number of mutants decreases if and only if we select the single mutant for death, i.e. $p^-(u) = 1/N$, for any node u. The number of mutants increases if and only if for death we select some node that neighbors u and then we select u for producing an offspring. Hence

$$p^+(u) = \sum_v p^+_{u,v},$$

where

$$p^+_{u,v} = \frac{1}{N} \cdot \frac{r \cdot w_{u,v}}{(r-1)\cdot w_{u,v} + \sum_{u'} w_{u',v}}$$

is the probability that v was selected for death and then u (the only mutant on G) was selected to place a copy of itself on v.

Now we bound $\mathrm{ep}^{\mathrm{Bd}}(G,r)$ in terms of $p^-(u)$ and $p^+(u)$. In the last step we use Jensen's inequality for a function $f(x) = 1/(1+x)$ which is convex on $x \in (0,\infty)$:

$$\mathrm{ep}^{\mathrm{dB}}(G,r) = \frac{1}{N}\sum_u \mathrm{ep}^{\mathrm{dB}}(u) \geq \frac{1}{N}\sum_u \frac{1}{1+\frac{p^+(u)}{p^-(u)}} \geq \frac{1}{1+\frac{1}{N}\sum_u \frac{p^+(u)}{p^-(u)}}.$$

Since $p^-(u) = 1/N$ for all u, the right-hand side simplifies and we get

$$\mathrm{ep}^{\mathrm{dB}}(G,r) \geq \frac{1}{1+\sum_u p^+(u)}.$$

In the rest, we find a tight upper bound on $\sum_u p^+(u)$. We first rewrite each $p^+(u)$ using $p^+_{u,v}$ and interchange the sums to get

$$\sum_u p^+(u) = \sum_u\sum_v p^+_{u,v} = \sum_v\sum_u p^+_{u,v}.$$

We focus on the inner sum. Fix a node v and denote by $s(v) = \sum_{u'} w_{u',v}$ the total weight of all edges incoming to v. Using the formula for $p^+_{u,v}$ we obtain

$$\sum_u p^+_{u,v} = \frac{1}{N}\sum_u \frac{r\cdot w_{u,v}}{(r-1)\cdot w_{u,v} + s(v)}.$$

We make three observations. First, the summation has at most $d_{in}(v)$ terms, where $d_{in}(v)$ is the number of incoming edges to v. Second, we have $\sum_u w_{u,v} = s(v)$. Third, for fixed $r > 0$ and any $s > 0$, the function $g(x) = \frac{r\cdot x}{(r-1)x+s}$ is concave on $x \in (0,s)$. Therefore, by another application of Jensen's inequality we can write

$$\sum_u p^+_{u,v} \leq \frac{1}{N}\cdot d_{in}(v) \cdot \frac{r\cdot \frac{s(v)}{d_{in}(v)}}{(r-1)\cdot\frac{s(v)}{d_{in}(v)} + s(v)} = \frac{1}{N}\cdot\frac{r\cdot d_{in}(v)}{r-1+d_{in}(v)},$$

Finally, summing up over v we obtain

$$\sum_u p^+(u) = \sum_v \sum_u p^+_{u,v} \le \frac{1}{N} \sum_v \frac{r \cdot d_{in}(v)}{r-1+d_{in}(v)} \le \frac{r \cdot d}{r-1+d},$$

where in the last step we yet again used Jensen's inequality, this time for the function $h(x) = \frac{r \cdot x}{r-1+x}$ that is concave on $x \in (0, \infty)$, and the fact that the average in-degree of a graph is the same as its average out-degree.

We conclude by observing that this upper bound on $\sum_u p^+(u)$ yields

$$\mathrm{ep}^{\mathrm{dB}}(G, r) \ge \frac{1}{1 + \sum_u p^+(u)} \ge \frac{1}{1 + \frac{r \cdot d}{r-1+d}} = \frac{d+r-1}{dr+d+r-1},$$

hence

$$\mathrm{fp}^{\mathrm{dB}}(G, r) \le 1 - \mathrm{ep}^{\mathrm{dB}}(G, r) \le \frac{d \cdot r}{d \cdot r + d + r - 1}$$

as desired □

With the lemma at hand, we can prove the first two Theorems.

Theorem 1 (All dB amplifiers are transient)**.** *Fix a non-complete graph G_N (possibly with directed and/or weighted edges). Then there exists $r^\star > 1$ such that for all $r > r^\star$ we have $\mathrm{fp}^{\mathrm{dB}}(G_N, r) < \mathrm{fp}^{\mathrm{dB}}(K_N, r)$, where K_N is the complete graph on N vertices. In particular, we can take $r^\star = 2N^2$.*

Proof. Recall that

$$\mathrm{fp}^{\mathrm{dB}}(K_N) = (1 - 1/N) \frac{1 - 1/r}{1 - 1/r^{N-1}} \ge (1 - 1/N)(1 - 1/r),$$

hence

$$\mathrm{ep}^{\mathrm{dB}}(K_N) \le \frac{N+r-1}{Nr}.$$

Using Lemma 1, it suffices to show that for all sufficiently large r we have

$$\frac{d+r-1}{dr+d+r-1} > \frac{N+r-1}{Nr}$$

which, after clearing the denominators, is equivalent to

$$r^2 (N-1-d) - 2r(N-1) - (d-1)(N-1) > 0.$$

Since G is not complete, $d < N-1$ (a strict inequality), hence the coefficient by r^2 is positive and the inequality holds for all sufficiently large r.

In particular, it is straightforward to check that $r = 2N^2$ is large enough: If G misses at least one edge then $d \le N - 1 - \frac{1}{N}$ hence for $r \ge 2N^2$ the right-hand side is at least

$$(2N^2)^2 \cdot \frac{1}{N} - 4N^2(N-1) - N^2 = 3N^2 > 0.$$

□

Theorem 2 (All dB amplifiers are bounded)**.** *Fix $r > 1$. Then for any graph G_N (possibly with directed and/or weighted edges) we have* $\mathrm{fp}^{\mathrm{dB}}(G_N, r) \le 1 - \frac{1}{r+1}$.

Proof. Using Lemma 1, it suffices to check that

$$\frac{d+r-1}{dr+d+r-1} \ge \frac{1}{r+1}$$

which, after clearing the denominators, is equivalent to $r(r-1) \ge 0$. The equality holds for $r = 1$. $\square$

4.2 Theorems on δ-dB updating

In order to prove Theorems 4 and 3 we first use a similar technique as before to establish an analogue of Lemma 1 that applies to δ-dB updating.

Lemma 2. Fix $r > 1$ and let G be a graph (possibly with directed and/or weighted edges) with average out-degree d. Then

$$\mathrm{ep}^{\delta}(G, r) \ge \frac{1}{1 + \frac{dr}{d+r-1} + \frac{1-\delta}{\delta} \cdot \frac{Nr}{N+r-1}}.$$

Proof. Denote the initial mutant node by u and, as in Lemma 1, let $p^-(u)$ (resp. $p^+(u)$) be the probability that after a single step of δ-dB updating, the number of mutants in the population decreases (resp. increases).

The values $p^-(u)$ and $p^+(u)$ are weighted averages of the corresponding values under (pure) dB and Bd updating, with weights δ, $1-\delta$. That is,

$$p^-(u) = \ \delta \cdot \frac{1}{N} \quad + \quad (1-\delta) \cdot \sum_t \frac{1}{N+r-1} \cdot \frac{w_{t,u}}{\sum_{u'} w_{t,u'}}$$

and, using the notation $p^+_{u,v}$ from Lemma 1,

$$p^+(u) = \ \delta \cdot \sum_v p^+_{u,v} \quad + \quad (1-\delta) \cdot \frac{r}{N+r-1}.$$

As in Lemma 1, we get

$$\mathrm{ep}^{\delta}(G, r) \ge \frac{1}{1 + \frac{1}{N}\sum_u \frac{p^+(u)}{p^-(u)}}.$$

For each fixed u, we bound $p^-(u)$ from below by ignoring the whole Bd-contribution. We get $p^-(u) \ge \frac{\delta}{N}$ which yields

$$\mathrm{ep}^{\delta}(G, r) \ge \frac{1}{1 + \frac{1}{\delta}\sum_u p^+(u)}$$

and it remains to bound $\sum_u p^+(u)$ from above. In $\sum_u p^+(u)$, the total Bd-contribution (summed over u) equals $(1-\delta)\frac{Nr}{N+r-1}$ and, as in Lemma 1, the total dB-contribution is at most $\delta \cdot \sum_u \sum_v p^+_{u,v} \le \delta \cdot \frac{rd}{r-1+d}$. In total, this yields

$$\mathrm{ep}^{\delta}(G, r) \ge \frac{1}{1 + \frac{dr}{d+r-1} + \frac{1-\delta}{\delta} \cdot \frac{Nr}{N+r-1}}$$

as desired. $\square$

Using Lemma 2 we present proofs of Theorems 3 and 4 from the main text.

Theorem 3 (All δ-dB amplifiers are transient)**.** *Fix a non-complete graph G on N vertices (possibly with directed and/or weighted edges) and $\delta \in (0,1]$. Then there exists $r^\star > 1$ such that for all $r > r^\star$ we have $\mathrm{fp}^\delta(G,r) < \widehat{\mathrm{fp}}^\delta(K_N,r)$, where K_N is the complete graph on N vertices.*

Proof. Let d be the average in-degree of G. Since G is not complete, we have $d < N-1$ (a strict inequality).

As in the proof of Theorem 1, recall that $\mathrm{ep}^{\mathrm{dB}}(K_N,r) \le \frac{N+r-1}{Nr}$. Moreover, $\mathrm{fp}^{\mathrm{Bd}}(K_N,r) = \frac{1-1/r}{1-1/r^N} \ge 1-1/r$, hence $\mathrm{ep}^{\mathrm{Bd}}(K_N,r) \le \frac{1}{r}$. This yields

$$1-\widehat{\mathrm{fp}}^\delta(K_N,r) = \widehat{\mathrm{ep}}^\delta(K_N,r) = \delta\cdot \mathrm{ep}^{\mathrm{dB}}(K_N,r) + (1-\delta)\,\mathrm{ep}^{\mathrm{Bd}}(K_N,r) \le \frac{1}{r} + \delta\cdot\frac{r-1}{Nr}$$

and by Lemma 2 it suffices to show that for all sufficiently large r we have

$$\frac{1}{1+\frac{dr}{d+r-1}+\frac{1-\delta}{\delta}\cdot\frac{Nr}{N+r-1}} \ge \frac{1}{r}+\delta\cdot\frac{r-1}{Nr},$$

Since N, d and δ are all fixed, we can consider both sides as functions of r. As $r\to\infty$, the left-hand side tends to $\frac{1}{1+d+\frac{1-\delta}{\delta}N}$ while the right-hand side tends to $\frac{\delta}{N}$. In order to conclude, it suffices to show strict inequality between the respective limits:

$$\frac{1}{1+d+\frac{1-\delta}{\delta}N} > \frac{\delta}{N}.$$

After clearing the denominators, this is equivalent to $\delta(N-1-d) > 0$ which indeed holds for any $\delta > 0$ and any non-complete graph K_N. ☐

Theorem 4 (All δ-dB amplifiers are at most linear)**.** *Fix $r > 1$ and $\delta \in (0,1]$. Then for any graph G (possibly with directed and/or weighted edges) we have $\mathrm{fp}^\delta(G,r) \le 1-\frac{1}{(r/\delta)+1}$.*

Proof. Since $d \le N-1 < N$ and $r>1$, we have

$$\frac{dr}{d+r-1} < \frac{Nr}{N+r-1},$$

hence Lemma 2 gives

$$\mathrm{ep}^\delta(G,r) \ge \frac{1}{1+\frac{dr}{d+r-1}+\frac{1-\delta}{\delta}\cdot\frac{Nr}{N+r-1}} > \frac{1}{1+\frac{1}{\delta}\cdot\frac{Nr}{N+r-1}} \ge \frac{\delta}{r+\delta} = \frac{1}{(r/\delta)+1},$$

where the last inequality is equivalent to $\delta\cdot r(r-1)\ge 0$ after clearing the denominators. The result follows. ☐

5 Further directions

Here we list several interesting open questions.

1. **Unweighted transient amplifiers under dB updating.** Weighted transient amplifiers for dB updating have been constructed in a companion work [4]. Do there exist transient amplifiers among unweighted graphs?

2. **Towards universal amplification under dB updating.** The weighted transient amplifiers constructed in the companion work [4] amplify for r less than a golden ratio $\phi = \frac{1}{2}(1+\sqrt{5}) \doteq 1.618$. Does there exist a graph that amplifies for $r > \phi$? If so, does for every $r^\star$ exist a graph that amplifies for all $r \in (1, r^\star)$? If so, does there exist a universal amplifier for dB updating? Theorem 1 implies that if so, it has to be a weighted version of a complete graph.

3. **Towards universal amplification under δ-dB updating.** How do the answers change under δ-dB updating instead of (pure) dB updating? Specifically, do large complete Bipartite graphs amplify on arbitrarily large intervals $(1, r^\star)$, provided that δ is small enough?

4. **Well-mixed populations with δ-updating.** Is there a simple formula for fixation probability on a complete graph under δ-dB updating for $\delta \in (0, 1)$?

5. **Monotonicity in δ.** Is $\mathrm{fp}^{\mathrm{dB}}(G_N, r) < \mathrm{fp}^{\mathrm{Bd}}(G_N, r)$ for any fixed graph G and any fixed $r > 1$? If so, is $\mathrm{fp}^{\delta}(G_N, r)$ a decreasing function of δ, for any fixed graph G and any fixed $r > 1$?

6. **Optimal graph for a given r.** For fixed $r > 1$, what is the highest possible fixation probability $\mathrm{fp}^{\mathrm{dB}}(G, r)$, attained by any graph G? Theorem 2 states that $\mathrm{fp}^{\mathrm{dB}}(G, r) \le 1 - 1/(r+1)$ for any fixed $r > 1$ and any graph G. The bound is attained for $r = 1$ due to K_2 and is relatively tight for $r \to \infty$ due to large Complete graphs which give $\mathrm{fp}^{\mathrm{dB}}(K_N, r) \to_{N\to\infty} 1 - 1/r$ (see Fig A in S1 Text). Are those graphs optimal? Or does there exist $r > 1$ and a graph G (of any size) such that $\mathrm{fp}^{\mathrm{dB}}(G, r) > \max\{\frac{1}{2}, 1 - \frac{1}{r}\}$?

7. **Regime $r < 1$.** Throughout this work we exclusively deal with regime $r > 1$. For $r < 1$, a population structure is an r-amplifier if it decreases the fixation probability of (disadvatageous) mutants. Do there exist graphs that are r-amplifiers under dB updating for all $r \in (0, 1)$?

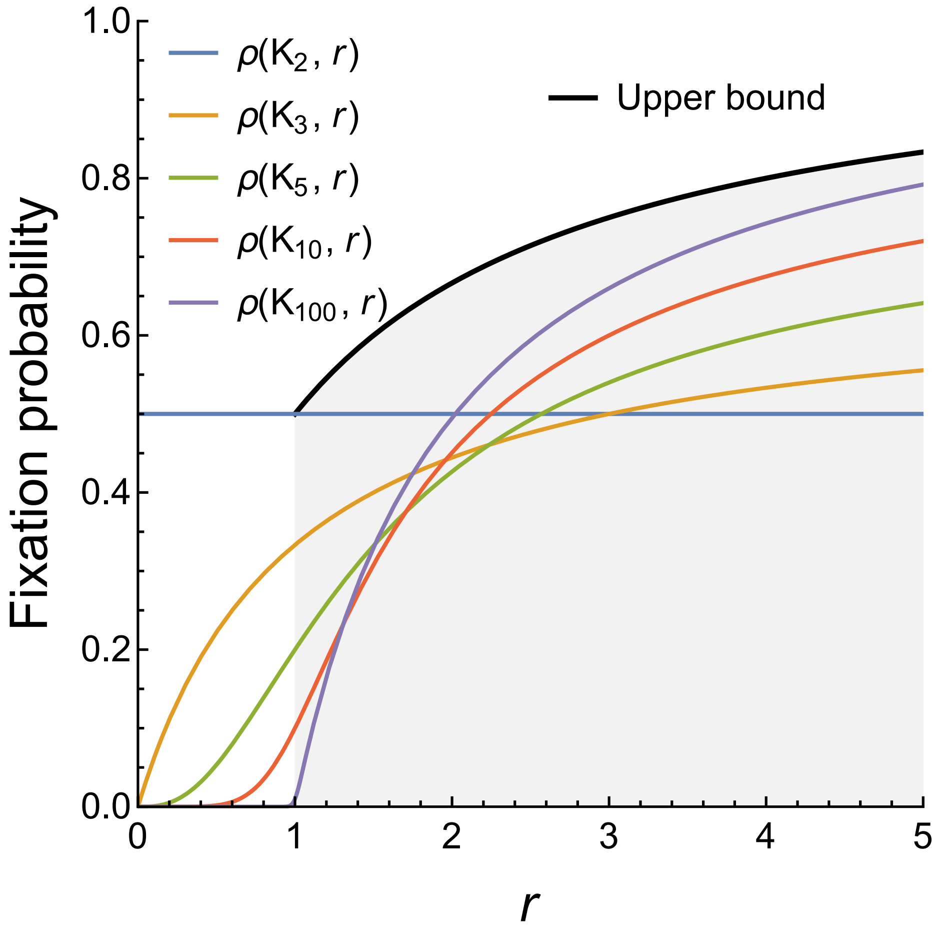

Fig A. **Additional figure: Tightness of the upper bound.** We consider Complete graphs of sizes $N \in \{2, 3, 5, 10, 100\}$ under dB updating. The fixation probability is always below the upper bound given by Theorem 2. For $r = 1$ the bound precisely matches the fixation probability on K_2. For large r, the bound is relatively tight with respect to large Complete graphs.

References

[1] Kaveh K, Komarova NL, Kohandel M. The duality of spatial death–birth and birth–death processes and limitations of the isothermal theorem. Royal Society open science. 2015;2(4):140465.

[2] Lieberman E, Hauert C, Nowak MA. Evolutionary dynamics on graphs. Nature. 2005;433(7023):312.

[3] Adlam B, Chatterjee K, Nowak M. Amplifiers of selection. Proc R Soc A Math Phys Eng Sci. 2015;471:20150114.

[4] Allen B, et al. Transient amplifiers of selection and reducers of fixation for death-Birth updating on graphs. 2019;.
